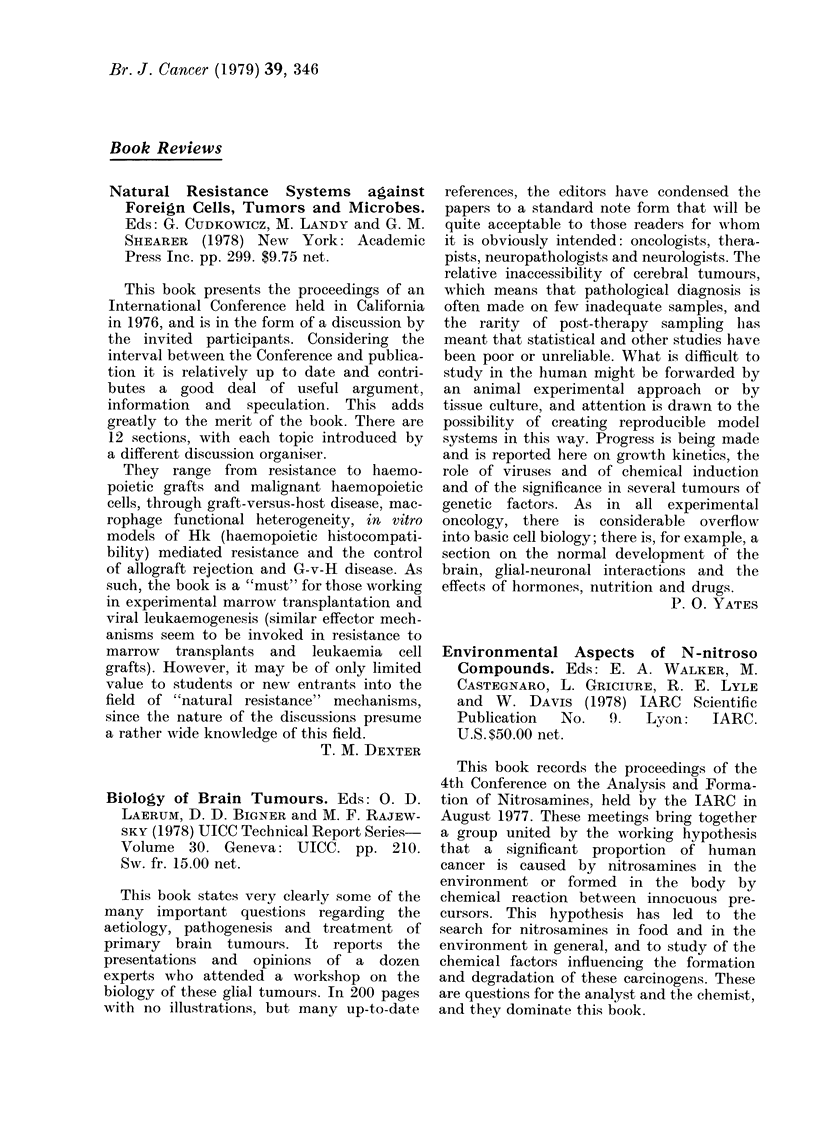# Biology of Brain Tumours

**Published:** 1979-03

**Authors:** P. O. Yates


**Biology of Brain Tumours.** Eds: O. D. Laerum, D. D. Bigner and M. F. Rajewsky (1978) UICC Technical Report Series—Volume 30. Geneva: UICC. pp. 210. Sw. fr. 15.00 net.

This book states very clearly some of the many important questions regarding the aetiology, pathogenesis and treatment of primary brain tumours. It reports the presentations and opinions of a dozen experts who attended a workshop on the biology of these glial tumours. In 200 pages with no illustrations, but many up-to-date references, the editors have condensed the papers to a standard note form that will be quite acceptable to those readers for whom it is obviously intended: oncologists, therapists, neuropathologists and neurologists. The relative inaccessibility of cerebral tumours, which means that pathological diagnosis is often made on few inadequate samples, and the rarity of post-therapy sampling has meant that statistical and other studies have been poor or unreliable. What is difficult to study in the human might be forwarded by an animal experimental approach or by tissue culture, and attention is drawn to the possibility of creating reproducible model systems in this way. Progress is being made and is reported here on growth kinetics, the role of viruses and of chemical induction and of the significance in several tumours of genetic factors. As in all experimental oncology, there is considerable overflow into basic cell biology; there is, for example, a section on the normal development of the brain, glial-neuronal interactions and the effects of hormones, nutrition and drugs.

P. O. Yates